# Supplementary material for: Use of medicinal plants for COVID-19 prevention and respiratory symptom treatment during the pandemic in Cusco, Peru: A cross-sectional survey
Source: PLoS One. 2021 Sep 22;16(9):e0257165. doi: 10.1371/journal.pone.0257165 (PMC8457479; doi:10.1371/journal.pone.0257165)
Supplement: S1 Annex — (DOCX) [file pone.0257165.s001.docx]

**ANNEX 1.** Survey to assess the use of medicinal plants in the prevention and treatment of respiratory symptoms during the COVID-19 pandemic.

**Demographic data**

1. **Sex:** Male ( ) Female ( )
2. **Age:** _________ (years)
3. **Education level:** No education ( ) Primary ( ) Secondary ( ) Technical ( ) University ( )
4. **Occupation or professional activity:** Housewife ( ) Self-employed ( ) Public sector ( ) Private sector ( ) Student ( ) Other ( )
5. **District in Cusco where you live:** Cusco ( ) San Jerónimo ( ) San Sebastián ( ) Santiago ( ) Wanchaq ( )
6. **Do you use medicinal plants to prevent respiratory symptoms related to COVID-19 during the pandemic?** Yes ( ) No ( )
7. **Do you use medicinal plants to treat respiratory symptoms related to COVID-19 during the pandemic?** Yes ( ) No ( )
8. **Have you been diagnosed with COVID-19?** Yes ( ) No ( )
9. **Have a family member or friend been diagnosed with COVID-19?** Yes ( ) No ( )
10. **Which medicinal plan have you used to prevent or treat respiratory symptoms related to COVID-19 during the pandemic?**

| **Medicinal plant** | **Yes** | **No** |
| --- | --- | --- |
| Eucalyptus |  |  |
| Ginger |  |  |
| Garlic |  |  |
| Coca |  |  |
| Muña |  |  |
| Matico |  |  |
| Chamomile |  |  |
| Rosemary |  |  |
| Oregano |  |  |
| Lemon balm |  |  |
| Geranium |  |  |
| Thyme |  |  |
| Panty |  |  |
| Keto-keto |  |  |
| Sage |  |  |
| Wira Wira |  |  |
| Asmachilca |  |  |

1. **What symptom(s) did you use the above-mentioned plants for?**

| **Medicinal plant** | **Malaise** | **Cough** | **Headache** | **Sore throat** | **Fever** | **Other** |
| --- | --- | --- | --- | --- | --- | --- |
| Eucalyptus |  |  |  |  |  |  |
| Ginger |  |  |  |  |  |  |
| Garlic |  |  |  |  |  |  |
| Coca |  |  |  |  |  |  |
| Muña |  |  |  |  |  |  |
| Matico |  |  |  |  |  |  |
| Chamomile |  |  |  |  |  |  |
| Rosemary |  |  |  |  |  |  |
| Oregano |  |  |  |  |  |  |
| Lemon balm |  |  |  |  |  |  |
| Geranium |  |  |  |  |  |  |
| Thyme |  |  |  |  |  |  |
| Panty |  |  |  |  |  |  |
| Keto-keto |  |  |  |  |  |  |
| Sage |  |  |  |  |  |  |
| Wira Wira |  |  |  |  |  |  |
| Asmachilca |  |  |  |  |  |  |
