# Supplementary material for: Use of medicinal plants for COVID-19 prevention and respiratory symptom treatment during the pandemic in Cusco, Peru: A cross-sectional survey
Source: PLoS One. 2021 Sep 22;16(9):e0257165. doi: 10.1371/journal.pone.0257165 (PMC8457479; doi:10.1371/journal.pone.0257165)
Supplement: S2 Annex — (DOCX) [file pone.0257165.s002.docx]

**ANNEX 2.** Survey to assess the use of medicinal plants in the prevention and treatment of respiratory symptoms during the COVID-19 pandemic in Spanish, the original language.

**Información demográfica**

1. **Sexo:** Hombre ( ) Mujer ( )
2. **Edad:** _________ (años)
3. **Nivel educativo:** No educación ( ) Primaria ( ) Secundaria ( ) Técnico ( ) Universitario ( )
4. **Ocupación o actividad profesional:** Ama de casa ( ) Autónomo ( ) Sector público ( ) Sector privado ( ) Estudiante ( ) Otro ( )
5. **Distrito en Cusco en el que vive:** Cusco ( ) San Jerónimo ( ) San Sebastián ( ) Santiago ( ) Wanchaq ( )
6. **¿Ha usado plantas medicinales para prevenir los síntomas respiratorios relacionados con la COVID-19 durante la pandemia?** Sí ( ) No ( )
7. **¿Ha usado plantas medicinales para tratar los síntomas respiratorios relacionados con la COVID-19 durante la pandemia?** Sí ( ) No ( )
8. **¿Ha sido diagnosticado con COVID-19?** Sí ( ) No ( )
9. **¿Algún familiar o amigo suyo ha sido diagnosticado con COVID-19?** Sí ( )

No ( )

1. **¿Cuál de las siguientes plantas medicinales ha usado para prevenir o tratar los síntomas respiratorios relacionados con la COVID-19 durante la pandemia?**

| **Planta medicinal** | **Sí** | **No** |
| --- | --- | --- |
| Eucalipto |  |  |
| Jengibre (Kión) |  |  |
| Ajo |  |  |
| Coca |  |  |
| Muña |  |  |
| Matico |  |  |
| Manzanilla |  |  |
| Romero |  |  |
| Orégano |  |  |
| Toronjil |  |  |
| Geranio |  |  |
| Tomillo |  |  |
| Panty |  |  |
| Keto-keto |  |  |
| Salvia |  |  |
| Wira Wira |  |  |
| Asmachilca |  |  |

1. **¿Por cuál(es) síntoma(s) usó las plantas medicinales mencionadas arriba?**

| **Planta medicinal** | **Malestar general** | **Tos** | **Dolor de cabeza** | **Dolor de garganta** | **Fiebre** | **Otro** |
| --- | --- | --- | --- | --- | --- | --- |
| Eucalipto |  |  |  |  |  |  |
| Jengibre (Kión) |  |  |  |  |  |  |
| Ajo |  |  |  |  |  |  |
| Coca |  |  |  |  |  |  |
| Muña |  |  |  |  |  |  |
| Matico |  |  |  |  |  |  |
| Manzanilla |  |  |  |  |  |  |
| Romero |  |  |  |  |  |  |
| Orégano |  |  |  |  |  |  |
| Toronjil |  |  |  |  |  |  |
| Geranio |  |  |  |  |  |  |
| Tomillo |  |  |  |  |  |  |
| Panty |  |  |  |  |  |  |
| Keto-keto |  |  |  |  |  |  |
| Salvia |  |  |  |  |  |  |
| Wira Wira |  |  |  |  |  |  |
| Asmachilca |  |  |  |  |  |  |
