# Supplementary material for: Use of medicinal plants for COVID-19 prevention and respiratory symptom treatment during the pandemic in Cusco, Peru: A cross-sectional survey
Source: PLoS One. 2021 Sep 22;16(9):e0257165. doi: 10.1371/journal.pone.0257165 (PMC8457479; doi:10.1371/journal.pone.0257165)
Supplement: Attachment — Submitted filename: Review report_Plos One.docx [file pone.0257165.s003.docx]

**Review report**

**GENERAL COMMENTS**

- Language and style should be checked and improved.
- Exact botanical names should be provided ([www.theplantlist.org](http://www.theplantlist.org) )

In Material and methods. What were the criteria used to include or exclude participants after the web-based investigation”?

- A diagram about the design of the study would add an important value to the paper. Please insert the number of participants in each step.
- How the authors calculated the sample? I need more details to judge the process.
- The selection seems to be biased. I need the exact process of participants’ selection.
- In the study protocol, participants were asked to select from a list of selected 17 medicinal species. This seems to be a serious bias. The authors should clarify and justify this approach.
- In the section results and discussion, replace the names of plants by their scientific names.
- For the mentioned species, important details are missing (parts used, administration routes, preparation, …..). This important information should be added.

**SPECIFIC COMMENTS**

**Section. Introduction.**

**The paragraph “**The idiosyncrasy of the population has also focused on the use of medicinal plants, natural products …………….. in the context of the COVID19 pandemic.”

Remove this paragraph and replace it with an overview about recent original or review papers discussing the use of medicinal plants to treat COVID-19.

**Section. M&M.**

“The respondents were asked to indicate if during the COVID-19 lockdown …………….. Center of Disease Control and Prevention (CDC) (45)”

This paragraph should be removed since it reports more details atht can be considered of less importance for the reader.

“The respondents were asked to select from a **list of 17 selected medicinal plants** the ones they have used to prevent or treat COVID-19 related respiratory symptoms.”

Why the authors asked the participants to select from a pre-established list of species? It seems that this can be a serious bias.

**Section. Results**

The associations between gender and different parameters should be discussed carefully since female were predominant. It’d be an effect of dominance and not a real effect of gender.

Page 14. “Therefore, it could be observed that in general the respondents preferably used medicinal plants as treatment of respiratory symptoms.”

I think this sentence can lead the reader to a wrong conclusion. This should be removed and discussed in the discussion section.

**Section. Discussion**

The most cited species should be further discussed and their use against COVID-19 should justified (bioactive compounds, previous *in vitro* or *in vivo* studies, ethnobotanical investigations especially in the region reporting their use to treat respiratory diseases, …).

**Page 14.** “As shown on Table 3, the respondents **mentioned** the use of **17 medicinal** plants for the treatment of COVID-19 related respiratory symptoms.”

By reading the study design, this statement is not true. Participants reported the use form a preselected 17 medicinal species!!!!!!
